# Draft Genome Sequence of Erysipelothrix rhusiopathiae, Isolated from a Canine Case of Diskospondylitis

**DOI:** 10.1128/MRA.00592-20

**Published:** 2020-06-25

**Authors:** Sara V. Little, Andrew E. Hillhouse, Sara D. Lawhon

**Affiliations:** a Department of Veterinary Pathobiology, College of Veterinary Medicine & Biomedical Sciences, Texas A&M University, College Station, Texas, USA; b Texas A&M Institute for Genome Sciences and Society, College of Veterinary Medicine & Biomedical Sciences, Texas A&M University, College Station, Texas, USA; University of Rochester School of Medicine and Dentistry

## Abstract

This is the draft genome of an Erysipelothrix rhusiopathiae strain isolated from the blood of a canine. Initial 16S ribosomal DNA amplification identified the isolate as belonging to the *Erysipelothrix* genus but could not elucidate the species due to previous misidentification of *E. rhusiopathiae* and E. tonsillarum. The species identification was confirmed by whole-genome sequencing.

## ANNOUNCEMENT

*Erysipelothrix* species are Gram-positive coccobacilli. The most common species is *E*. *rhusiopathiae*, which causes septicemia, joint infection, and skin lesions in a variety of animal species, primarily in pigs (1). In dogs, there are a few case reports that document bacteremia, septic polyarthritis, endocarditis, and infection of the aortic valve (2–11). Previous work has suggested that some isolates from dogs with endocarditis are E. tonsillarum rather than E. rhusiopathiae (12).

Here, we present the draft genome of Erysipelothrix rhusiopathiae strain 268691, isolated from a 2.5-year-old male Great Dane canine that presented for lumbar pain and was diagnosed with diskospondylitis. Amplification of the 16S ribosomal DNA as previously described (13) identified the bacterium as belonging to the *Erysipelothrix* genus but could not identify the species.

Three independent blood samples were collected and cultured by inoculating the blood into a commercial blood culture system (Bactec Plus aerobic/F culture vials; BD Franklin Lakes, NJ). Subcultures were plated at 24 h, 48 h, and 7 days onto Trypticase soy agar supplemented with 5% sheep’s blood (BAP). The isolates were stored at −80°C in brucella broth supplemented with 10% glycerol and revived for sequencing by inoculating an aliquot of the frozen bacteria onto a BAP. An isolated colony was used to inoculate a 5-ml culture of Trypticase soy broth, which was incubated overnight. A 1-ml aliquot of this culture was used for DNA isolation. All cultures were incubated at 35°C ± 2°C in an atmosphere supplemented with 5% CO_2_.

For the subsequent procedures, default parameters and manufacturer’s protocols were used unless stated otherwise. Genomic DNA was extracted from 1-ml aliquots of each isolate that were pelleted and subsequently lysed in a Qiagen TissueLyser using Macherey-Nagel bead tubes (type B) and lysis buffer from the NucleoMag tissue DNA kit. DNA was isolated using a commercial kit following the manufacturer’s protocol (Macherey-Nagel). Prior to sequencing, the DNA quality was verified using a genomic DNA TapeStation run (Agilent). Illumina libraries were prepared using the Illumina Nextera DNA Flex library preparation kit. An Illumina MiSeq v2 2 × 250-bp kit was used for sequencing. The sequencing data were uploaded onto Illumina’s BaseSpace for run monitoring, FASTQ generation, demultiplexing, and adapter trimming.

Sequencing resulted in 2,853,326 paired-end reads of 251 bp, which is approximately 400× coverage, with an *N*_50_ value of 303,535 bp. These reads were assembled using SPAdes v3.13.0 with the “careful” parameter (14). The resultant assembly was 1,697,258 bp long and had 77 contigs and a GC content of 37.41%. Annotation was completed using PGAP using the default parameters during submission to the NCBI Genome Submission Portal. The genome was analyzed for completeness using BUSCO (*Firmicutes* database) with a resultant score of 84.9%—potentially a lower score due to this species having one of the smallest genomes in the phylum *Firmicutes* (∼1,700,000 bp) and missing many typical orthologs for cell wall genes, fatty acid biosynthesis pathways, and amino acid biosynthesis genes (15). Species identification was confirmed using ribosomal multilocus sequence typing (rMLST) (16), with 100% support from the database, which included comparison to *E. tonsillarum*, as well as NCBI’s average nucleotide identity analysis (17).

### Data availability.

This whole-genome shotgun sequencing project has been deposited at DDBJ/ENA/GenBank under the accession number JAAAMP000000000; the raw MiSeq reads are available under SRA accession number SRR10850371. This announcement represents the first version of the genome.
